# ROLES OF GENDER AND SOCIAL MEDIA USE: MODERATING THE RELATIONSHIP BETWEEN FRUSTRATION AND DEPRESSIVE SYMPTOMS

**DOI:** 10.1093/geroni/igae098.3437

**Published:** 2024-12-31

**Authors:** Vivian Weiqun Lou, Clio Yuen Man Cheng

**Affiliations:** The University of Hong Kong, Hong Kong, China (People’s Republic); The University of Hong Kong, Hong Kong, China (People’s Republic)

## Abstract

Depressive symptoms among older adults pose a significant public health challenge, requiring thorough investigation of contributing factors. This study aimed to examine the role of gender and social media use as moderators in the relationship between frustration of basic psychological needs and depressive symptoms among young-olds (N = 686; Mage = 61.9 years; SD = 5.82), with implications for the development of targeted and tailored interventions. The regression model yielded statistical significance (F(6, 679) = 46.74, p < 0.001), accounting for 28.61% of the variance in depressive symptoms among young-olds. Notably, a significant interaction effect emerged between frustration and social media use (β = -0.042, p = 0.019). Young-olds who reported higher levels of frustration and increased social media use displayed elevated depressive symptoms, suggesting the potential detrimental impact of excessive social media consumption on mental well-being. Furthermore, the interaction effect between gender and social media use was also significant (β = 2.161, p < 0.001), indicating distinct group differences. Among males, a significant negative interaction effect was observed between frustration and social media use on depressive symptoms (β = -.099, p =.006). Conversely, no significant interaction effect between frustration and social media use was found among females (β = -.017, p =.392). Overall, these findings highlight the complex interplay of frustration, social media use, gender, and depressive symptoms among young-olds. Tailored interventions addressing the unique needs and vulnerabilities of different gender groups and promoting healthy social media use is crucial in reducing depressive symptoms in this population.

